# The aryl hydrocarbon receptor, CIITA and HLA-II: who is watching the watchdog?

**DOI:** 10.1186/s13046-026-03692-9

**Published:** 2026-04-10

**Authors:** Doriana Fruci, Patrizio Giacomini

**Affiliations:** 1 Department of Pediatric Hematology and Oncology, Bambino Gesù Children’s Hospital IRCCS, Rome, Italy; 2 UOSD Medicina di Precisione in Senologia, Fondazione Policlinico Universitario Agostino Gemelli IRCCS, Largo Agostino Gemelli, 8, Rome, 00168 Italy

**Keywords:** Aryl Hydrocarbon Receptor, CIITA, HLA class II, Environmental sensing, Antigen presentation

## Abstract

**Background:**

A recent paper by Jin et al. reveals an unexpected role of the Aryl Hydrocarbon Receptor (AHR), so far considered an environmental sensor. The authors demonstrate that AHR transactivates CIITA, thereby up-regulating antigen-presenting HLA-II molecules in cutaneous melanoma.

**Main body:**

In this commentary, we review the evidence supporting this original observation and discuss its potential biological implications.

**Conclusions:**

These findings suggest that the environmental exposome and HLA-II-mediated antigen presentation should be considered interconnected aspects of self-non-self-discrimination, with potentially important implications for tumor (immune) surveillance.

## Background

Thirty-three years after its seminal discovery by Steimle et al. [1], the Class II Major Histocompatibility Complex Transactivator (CIITA) remains the sole master transcriptional activator of antigen-presenting Human Leukocyte Antigen class II (HLA-II) molecules. CIITA expression is controlled by four lineage-specific promoters (pI to pIV), and functions as a molecular ‘watchdog’ that tightly controls HLA-II antigen presentation in space and time. Under physiological conditions, HLA-II expression is confined to professional antigen-presenting cells. Yet, unorthodox or aberrant HLA-II expression in non-lymphoid normal tissues and tumor cells (e.g. melanoma) has been documented over the past 5 decades [2, 3], perplexing generations of immunologists. The bottom line is that several CIITA promoters can be active in HLA-II-positive tumor cells; nevertheless, CIITA regulation is largely governed by epigenetic mechanisms and transcriptional (co)-repressors. Known positive regulators are almost exclusively interferon (IFN)-inducible (reviewed in [4]). To date, no specific upstream transacting factor has been clearly identified that drives constitutive CIITA/HLA-II expression, particularly in non-lymphoid lineages and tumors, including cutaneous melanoma. Thus, if CIITA is the watchdog of HLA-II expression, the most obvious open question is: who is watching the watchdog?

## Main text

Many researchers (including the authors of this commentary) would have anticipated that constitutive HLA-II expression might be controlled by lineage-specific and/or housekeeping transcriptional activators. While this possibility cannot be entirely excluded, in a recent issue of JECCR Jin and colleagues identify an unexpected regulator: the Aryl Hydrocarbon Receptor (AHR). They demonstrate that AHR controls constitutive HLA-II expression in several melanoma cells *via* CIITA [5]. This finding represents a genuine twist in the tale, and for several reasons.

AHR is a ubiquitously expressed receptor activated by a myriad of ligands derived from endogenous metabolism, diet, the microbiota, and the chemical exposome [6, 7]. By definition, AHR functions as an inducible environmental sensor and signal adaptor/integrator rather than as a classical housekeeping transcription factor. Upon ligand binding, AHR heterodimerizes with the Aryl Hydrocarbon Receptor Nuclear Translocator (ARNT), and binds Xenobiotic Response Elements (XRE) in the promoters of target genes, inducing programs involved in xenobiotic metabolism and detoxification [6, 7]. Canonical AHR targets include members of the Cytochrome P450 (CYP) superfamily, as well as Indoleamine 2,3-dioxygenase 1 (IDO1), an immunosuppressive checkpoint-like molecule [6, 7].

Jin et al. demonstrate that the AHR/ARNT complex binds an XRE-like element within the CIITA promoter. Remarkably, this XRE element resides in promoter pII, e.g. the only human-specific CIITA promoter, and until now the only one lacking a defined function. At first glance, at least three features of AHR signaling appear difficult to reconcile with CIITA/HLA-II biology. AHR signaling is ligand-dependent rather than constitutive, ubiquitous rather than tissue-restricted, and evolutionarily conserved, whereas the CIITA pII promoter is human-specific. Below, we argue that these apparent contradictions may at least in part be reconciled by considering 4 independent lines of evidence: ligand-dependent AHR conformations, tissue-specific distribution and signaling of AHR and its ligands, promoter preferences of CIITA pII, and AHR evolution.

Different AHR ligands are known to induce distinct receptor conformational changes, leading to specialized downstream signaling pathways [7]. In addition, several AHR functions are organ-specific, acquired during embryonic development, and maintained throughout adult life [6]. A compelling example of HLA-II being regulated by AHR through a tissue-specific pathway is provided by Jin and collaborators using FICZ (6-Formylindolo[2-b]carbazole). FICZ is a tryptophan-derived metabolite and potent AHR ligand selectively induced by UV light in the skin. Jin et al. demonstrate that FICZ robustly induces HLA-II expression in cutaneous melanoma cells. Consistent with tissue specificity, AHR activity is particularly prominent in so-called barrier tissues lining the outer body surfaces, e.g. skin, lung, and intestine [6, 7]. Interestingly, cells and histopathological structures in these tissues (melanocytes, bronchial glands, and the intestinal mucosa) represent preferential sites of HLA-II expression in humans, but not mice [3]. Accordingly, when the human HLA-II gene HLA-DRA is expressed under the control of its own promoter in a pII-less CIITA transgenic mouse background, its expression is selectively absent from human-specific sites [8]. These findings may help explain how a ubiquitous transactivator such as AHR can induce gene-specific and tissue-selective effects. Whether HLA-II expression in human non-lymphoid tissues or tumors reflects all or some of these regulatory mechanisms remains to be tested using additional ligands besides FICZ. One candidate of particular interest, not tested by Jin et al., is ITE (2-[1’H-indole-3’-carbonyl]-thiazole-4-carboxylic acid methyl ester), an endogenous AHR ligand with known immunomodulatory properties [9].

Further support for a human-specific AHR/HLA-II connection comes from evolutionary considerations. Recent evidence indicates that modern humans harbor a functional AHR polymorphism absent in extinct hominins [10]. This variant has been linked to enhanced tolerance to complex xenobiotics, such as Polycyclic Aromatic Hydrocarbons (PAH) generated by wood burning ([10] and reviewed therein). However, human migration, repeated dispersal events, and the acquisition of critical rudimentary technologies likely implied exposure not only to novel environmental chemicals and diets, but also to novel antigens and pathogens. From an evolutionarily perspective, a single signal integrator (AHR) simultaneously alerting for two major non-self-threats may provide a selective advantage. Co-evolution of a human-specific AHR variant together with CIITA/pII regulatory sequences would support a uniquely human ‘watchdog-of-watchdog’ relationship between AHR and CIITA.

The AHR/ARNT-CIITA-HLA-II axis therefore forges unexpected conceptual links among pollutant sensing, the chemical exposome, xenobiotic metabolism, CYP-mediated detoxification (notably, one CYP gene maps to the HLA region), tryptophan metabolism, the microbiota, and adaptive immune surveillance. Indeed, XRE elements in the promoters of CYP, IDO1, and now HLA-II, place respiratory toxicants, dietary metabolites and microbiota-derived indoles at the center of the immunological scene.

A vast literature supports both beneficial and detrimental roles of AHR and HLA-II in cancer and immunity, underscoring profound context-dependence of their effects [4, 6, 7, 9]. Extensive crosstalk with a number of canonical pathways including JAK-STAT, NF-κB, HIF-1α, and estrogen receptor, represents a major challenge for therapeutic exploitation in autoimmunity and cancer immunotherapy. AHR signaling appears to be predominantly tolerogenic on immune cells, including dendritic cells, regulatory T cells (Tregs), and type 1 regulatory T cells (Tr1), although a pro-inflammatory T CD4 helper switch (Th1 to Th17) is also possible.

An important question concerns the net effect of the multiple immune and non-immune pathways (including HLA-II) simultaneously triggered by the AHR. This may be addressed in the context of several ongoing clinical trials evaluating AHR antagonists [6], as well as real-life studies. For instance, PAHs such as benzopyrene bind AHR in the lung, triggering CYP1A1-mediated detoxification attempts that may paradoxically generate carcinogenic intermediates [7]. In this context, does AHR signaling also induce HLA-II expression in the bronchiolar epithelium? If so, does this exacerbate inflammation, or instead mitigate chronic tissue damage associated with Chronic Obstructive Pulmonary Disease (COPD) and lung cancer? Along the same line, does FICZ-mediated HLA-II induction in melanoma cells favor local anti-tumor immunity, or is it overridden by concomitant AHR-mediated IDO1 induction and checkpoint-mediated immunosuppression?

Jin et al. identify a composite gene-expression signature (HLA-II^high^/AHR-ARNT KO sig^low^) associated with favorable outcomes in melanoma and lung cancer. Whether this integrated signature provides superior prognostic power compared with its individual components remains an open and important question to be addressed in pre-clinical and clinical studies.

It is remarkable that, unlike IFN-mediated induction, AHR activation by FICZ elicits a slow but sustained HLA-II response peaking around day 6 and only gradually declining thereafter. This kinetic profile suggests that the AHR-CIITA-HLA-II axis may be evolutionarily tuned to support sub-acute or chronic immune responses, complementing the rapid and transient biological effects of classical immune cytokines.

**Fig. 1:**
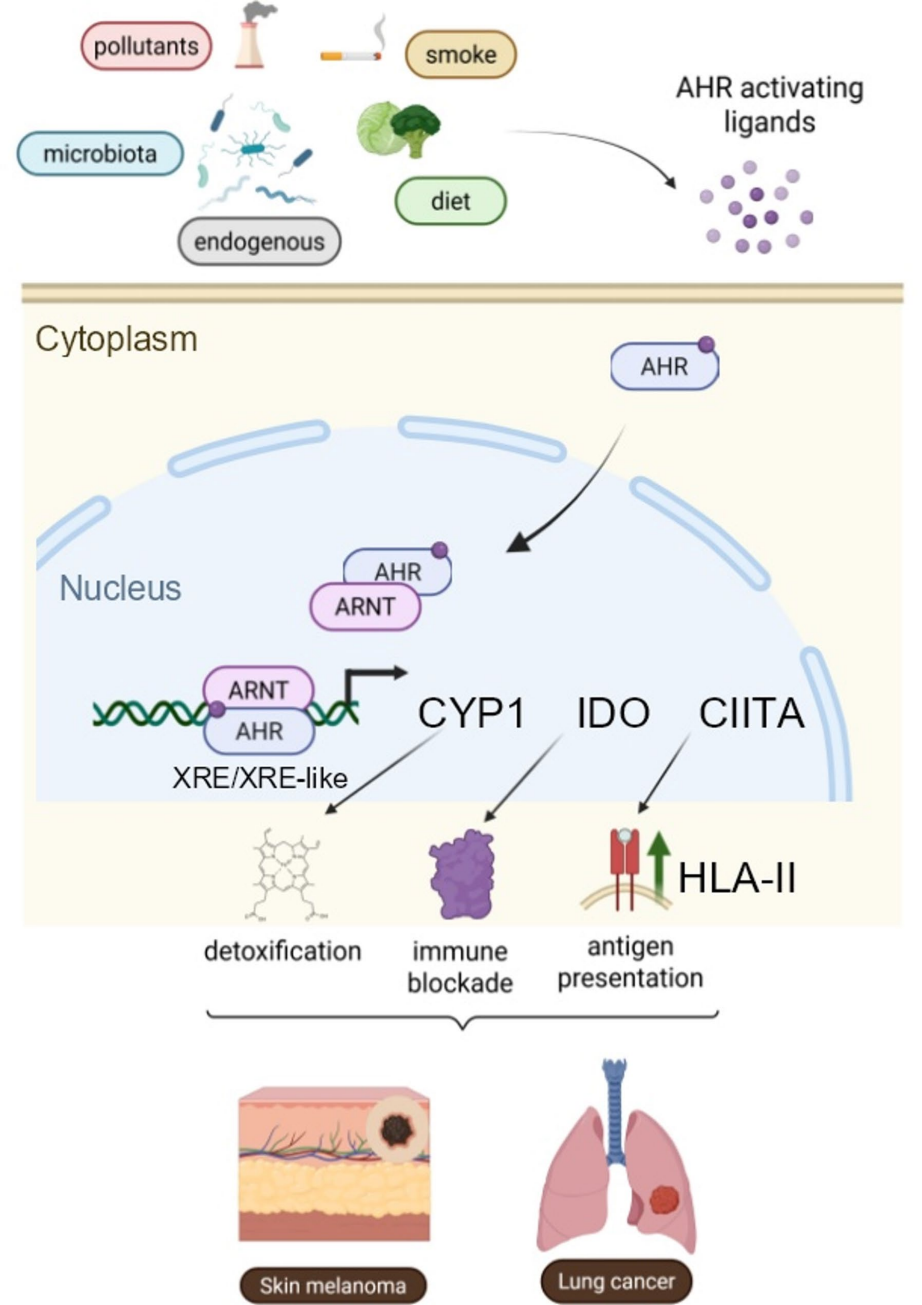
AHR bridges environmental and immune self-non-self-discrimination at barrier tissues. Xenobiotic sensing induces AHR/ARNT heterodimerization, and downstream signaling through shared XRE and XRE-like promoter elements. This triggers not only xenobiotic detoxification (CYP1A1/1B1) but also a minimum of two major immune sensing pathways: IDO enzymes (mainly immunosuppressive) and CIITA/HLA-II (possibly immunostimulatory). These may be seen as physiological functions in normal skin and lung. Their roles remain to be addressed when tumors (e.g. melanoma and lung cancer) arise at these body districts. Created by Biorender

Finally, Jin et al. report that AHR also modulates HLA class I (HLA-I) expression, albeit in a cell line-dependent and nuanced manner. These more limited effects are unsurprising, as HLA-I transcription is primarily controlled by NOD-like receptor family CARD domain containing 5 (NLRC5), a distant homolog of CIITA that often overrides CIITA-mediated effects. Both CIITA and NLRC5 are non-DNA-binding transcriptional activators that converge on related promoter architectures and share roles in inflammasome regulation [4]. In this broader context, the AHR/CIITA axis may link multiple adaptive and innate pathways involved in non-self-sensing (Fig. 1).

## Conclusions

If validated and extended by further studies, the findings reported by Jin and colleagues offer a striking example of how seemingly distal biological systems (e.g. environmental sensing and antigen presentation) can intersect to reshape our understanding of (tumor) immunology, driving research into uncharted territories.

## Data Availability

No datasets were generated or analysed during the current study.

## Abbreviations

AHR: Aryl Hydrocarbon Receptor
ARNT: Aryl Hydrocarbon Receptor Nuclear Translocator
CIITA: Class II Major Histocompatibility Complex Transactivator
COPD: Chronic Obstructive Pulmonary Disease
CYP: Cytochrome P450
FICZ: 6-Formylindolo[2-b]carbazole
HLA-I: Human Leukocyte Antigen class I
HLA-II: Human Leukocyte Antigen class II
IDO1: Indoleamine 2,3-dioxygenase 1
IFN: interferon
NLRC5: NOD-like receptor family CARD domain containing 5
PAH: Polycyclic Aromatic Hydrocarbons
XRE: Xenobiotic Response Elements
